# Early Detection and Prevention of Alzheimer’s Disease: Role of Oxidative Markers and Natural Antioxidants

**DOI:** 10.3389/fnagi.2020.00231

**Published:** 2020-07-27

**Authors:** Jamshed Arslan, Humaira Jamshed, Humaira Qureshi

**Affiliations:** ^1^Department of Basic Medical Sciences, Faculty of Pharmacy, Barrett Hodgson University, Karachi, Pakistan; ^2^Department of Integrated Sciences and Mathematics, Dhanani School of Science and Engineering, Habib University, Karachi, Pakistan

**Keywords:** reactive oxygen species, mild cognitive impairment, lipid peroxidation, tocopherols, polyphenols

## Abstract

Oxidative stress (OS) contributes to Alzheimer’s disease (AD) pathology. OS can be a result of increased reactive oxygen/nitrogen species, reduced antioxidants, oxidatively damaged molecules, and/or a combination of these factors. Scientific literature is scarce for the markers of OS-specific for detecting AD at an early stage. The first aim of the current review is to provide an overview of the potential OS markers in the brain, cerebrospinal fluid (CSF), blood and/or urine that can be used for early diagnosis of human AD. The reason for exploring OS markers is that the proposed antioxidant therapies against AD appear to start too late to be effective. The second aim is to evaluate the evidence for natural antioxidants currently proposed to prevent or treat AD symptoms. To address these two aims, we critically evaluated the studies on humans in which various OS markers for detecting AD at an early stage were presented. Non-invasive OS markers that can detect mild cognitive impairment (MCI) and AD at an early stage in humans with greater specificity and sensitivity are primarily related to lipid peroxidation. However, a combination of OS markers, family history, and other biochemical tests are needed to detect the disease early on. We also report that the long-term use of vitamins (vitamin E as in almonds) and polyphenol-rich foods (curcumin/curcuminoids of turmeric, ginkgo biloba, epigallocatechin-3-gallate in green tea) seem justified for ameliorating AD symptoms. Future research on humans is warranted to justify the use of natural antioxidants.

## Introduction

Alzheimer’s disease (AD) is the most common dementia of the elderly (Fattoretti et al., 2018). The discovery that memory decline often precedes other neuropathological signs of AD (Thomas et al., 2020) has ignited an interest in the pathology of AD, especially the translational state between normal aging and AD called mild cognitive impairment (MCI). Oxidative stress (OS) underlies MCI (Cervellati et al., 2014a; Di Domenico et al., 2016) and neurodegenerative diseases including AD (Dong et al., 2018). Various antioxidants have been suggested to prevent or even cure AD (Boasquivis et al., 2018; Mohamed et al., 2018; Popli et al., 2018). Major biomarkers of oxidative damage in AD have been identified (Smith et al., 1997; Nourooz-Zadeh et al., 1999; Lauderback et al., 2001; Halliwell, 2006; Dizdaroglu et al., 2015; Milne et al., 2015; Wang et al., 2015; Di Domenico et al., 2016, 2017; Dai et al., 2018; Ishii et al., 2018), but only a limited data on the usefulness of these biomarkers in the early detection of AD is available (Garcia-Blanco et al., 2017). Several antioxidants have been proposed for ameliorating oxidative damage in humans and non-human models of AD (Butterfield and Halliwell, 2019). Among these, some are preventive while others are touted to have a curative effect in AD. A discussion on the rigor of the evidence favoring the purported antioxidants in preventing or treating human AD is scarce.

Therefore, our objective is to highlight potential oxidative markers that can be used for early diagnosis of human AD and to evaluate the evidence for the natural antioxidants currently proposed to prevent or treat AD in humans. Primary research on OS and antioxidants in the context of human MCI and/or AD was analyzed. The studies on early detection of AD, preclinical AD, or MCI in humans in terms of oxidative damage and the research on antioxidants useful in these conditions were selected for review. It should be noted that the animal studies, the discussion on effects of any non-dietary intervention like exercise on MCI/AD (Suridjan et al., 2017), trials on synthetic compounds with antioxidant anti-AD potential like statins (Chu et al., 2018), data on novel or synthetic antioxidant supplements for AD (Tadokoro et al., 2020), OS-biometal association in MCI/AD (Balmus et al., 2017), and studies on patients with comorbidities (Zheng et al., 2016) were deemed outside the scope of this article.

## Oxidative Stress Markers for Early Detection of AD

Studies have reported various products derived from proteins, lipids, DNA, or RNA that indicate OS in the brain. For example, OS damage to the protein can be determined by measuring 3-nitrotyrosine, protein carbonyls, methionine sulfoxide or highly reactive aldehydes; lipid damage by determining isoprostanes and lipid and cyclic peroxides; DNA damage by estimating 8-hydroxy-deoxyguanosine (8OHdG); and RNA damage has been determined by measuring 8-hydroxyguanine (8OHG; Butterfield and Halliwell, 2019).

### Oxidative Damage to Proteins

Elevated levels of protein carbonyls and 3-nitrotyrosine in the MCI lymphocyte mitochondria (Sultana et al., 2013) and in the frontal cortex (Ansari and Scheff, 2010) and hippocampus of MCI and AD (Scheff et al., 2016) indicate that OS damage to proteins is an early sign of AD. Oxidative inactivation of several proteins in the hippocampus leads to the progression of AD from MCI (Butterfield et al., 2006a). The oxidatively modified proteins in the cerebrospinal fluid (CSF) of MCI, as determined by redox proteomics, remain oxidized in the disease progression to AD (Di Domenico et al., 2016). Both MCI and AD patients show increased plasma levels of advanced oxidation protein products (Chico et al., 2013). Increased carbonyl groups content in the plasma of early AD subjects have been reported (Puertas et al., 2012). Carbonyl proteins in the plasma can be roughly three times higher in MCI/AD relative to the age-matched healthy controls (Greilberger et al., 2010; Table 1).

**Table 1:** Oxidative markers for early detection of Alzheimer’s disease.

Author, year	Groups	Sample	Key result
Ansari and Scheff (2010)	HC = (*n* = 10) MCI (*n* = 8) Mild-to-moderate AD (*n* = 4) Late-stage AD (*n* = 9).	Brain tissue	Elevated levels of protein carbonyls and 3-nitrotyrosine in the frontal cortex of MCI and AD in a disease-dependent manner.
Arce-Varas et al. (2017)	HC (*n* = 44) MCI (*n* = 43) AD (*n* = 53)	Plasma and peripheral mononuclear cells	Decreased SOD is observed in MCI and AD, pointing to the importance of considering extracellular and intracellular blood compartments in evaluating oxidative stress
Baldeiras et al. (2010)	MCI (*n* = 70)	Serum	MDA levels have been correlated with the progression of MCI into AD
Cervellati et al. (2013)	HC (*n* = 99) MCI (*n* = 134) AD (*n* = 101)	Serum	High hydroperoxides levels associated with MCI and AD
Cervellati et al. (2014a)	HC (*n* = 118) MCI (*n* = 111) AD (*n* = 105)	Serum	High hydroperoxide levels associated with MCI and AD. Antioxidant capacity in AD and MCI is lower than that of HC
Cervellati et al. (2014b)	HC (*n* = 48) MCI (*n* = 103) AD (*n* = 89)	Serum	High hydroperoxides with low residual antioxidant capacity are more pronounced in MCI and AD as compared to HC.
Chico et al. (2013)	HC (*n* = 63) MCI (*n* = 34) AD (*n* = 85)	Plasma	Both MCI and AD patients have increased levels of advanced oxidation protein products. APOE4 carriers MCI have reduced plasma SOD activity relative to non-APOE4 carriers. Plasma reducing capacity AD < MCI < HC
Di Domenico et al. (2016)	HC (*n* = 6) MCI (*n* = 6) AD (*n* = 6)	CSF	Oxidatively modified proteins in the CSF of MCI remain oxidized in disease progression to AD
Du et al. (2019)	HC (*n* = 832) MCI (*n* = 113)	Serum	IMA is a potential biomarker for oxidative stress in MCI
Greilberger et al. (2010)	HC (*n* = 15) MCI (*n* = 6) AD (*n* = 10)	Plasma	Carbonyl proteins in plasma can be roughly three times higher in MCI/AD relative to HC.
Lopez et al. (2013)	HC (*n* = 33) MCI (*n* = 18) AD (*n* = 36)	Blood	MDA levels MCI > HC SOD activity AD < HC
Mangialasche et al. (2013)	HC (*n* = 86) MCI (*n* = 86) AD (*n* = 81)	Serum	Higher levels of gamma-tocopherol, beta-tocotrienol, total tocotrienols, and gamma-tocopherol/cholesterol ratio are associated with a lower risk of MCI or AD in the older adults
Martinez de Toda et al. (2019)	HC (*n* = 30) MCI (*n* = 20) AD (*n* = 20)	Blood	Higher TBARS and lower glutathione peroxidase and reductase activities in both sexes can be a marker for prodromal AD
Nunomura et al. (2012)	HC (*n* = 5) MCI (*n* = 6) AD (*n* = 5)	Brain tissue	Oxidized RNA nucleoside 8OHG in the neurons of the cerebral cortex is an age-associated phenomenon, but a more prominent RNA damage correlates with MCI and AD
Picco et al. (2014)	HC (*n* = 23) MCI (*n* = 28) AD (*n* = 34)	Brain and plasma	SOD activity and brain glucose metabolism AD < MCI < HC
Puertas et al. (2012)	HC (*n* = 46) MCI (*n* = 46)	Plasma	Carbonyl groups content, thiobarbituric acid reactive substances (index of lipid peroxidation) MCI > HC Plasma glutathione levels and antioxidant enzymes such as glutathione peroxidase, catalase, and superoxide dismutase (SOD) HC > MCI
Rita Cardoso et al. (2014)	HC (*n* = 29) MCI (*n* = 31) AD (*n* = 28)	Red blood cells and plasma	Antioxidant selenium levels HC > MCI > AD
Scheff et al. (2016)	HC (*n* = 48) MCI (*n* = 15)	Brain	Increased protein carbonyls, 4-hydroxynonenal and 3-nitrotyrosine in hippocampus enhances the likelihood of AD-like pathology
Sultana et al. (2013)	HC (*n* = 10) MCI (*n* = 12)	Blood	Elevated levels of protein carbonyls and 3-nitrotyrosine, and 4-hydroxy-2-nonenal-bound proteins in MCI lymphocyte mitochondria relative to HC
Torres et al. (2011)	HC (*n* = 26) MCI (*n* = 33) AD (*n* = 29)	Red blood cells and plasma	Plasma production of MDA and catalase activity AD > MCI > HC Glutathione reductase/glutathione peroxidase ratio HC > MCI > AD

*AD, Alzheimer’s disease; APOE4, Apolipoprotein E4; CSF, Cerebrospinal fluid; HC, Healthy control with no cognitive impairment; IMA, Ischemia-modified albumin; MCI, Mild cognitive impairment; MDA, malondialdehyde; 8OHG, 8-hydroxyguanosine; SOD, superoxide dismutase; TBARS, thiobarbituric acid-reactive substances*.

The specificity of plasma carbonyl proteins is still questionable since one cannot differentiate between AD and other dementias like vascular dementia based on carbonyl proteins alone (Polidori et al., 2004). Likewise, caloric restriction itself reduces oxidative damage to the brain proteins, measured by protein carbonyl levels (Forster et al., 2000). Further investigations are warranted that record patient’s dietary habits whilst evaluating the link between plasma carbonyl proteins and early AD.

### Role of Lipid Peroxidation

The plasma, CSF, and urine of MCI subjects exhibit higher levels of isoprostane 8,12-iso-iPF(2alpha)-VI, a marker of *in vivo* lipid peroxidation, as compared to cognitively normal elderly controls (Pratico et al., 2002). Plasma and whole blood levels of thiobarbituric acid reactive substances, an index of lipid peroxidation, are likewise high in early AD (Puertas et al., 2012; Martinez de Toda et al., 2019). Lipid hydroperoxides are the unstable products of lipid peroxidation that undergo non-enzymatic decomposition to generate aldehydes like malondialdehyde (MDA) and 4-hydroxynonenal (4-HNE); latter form covalent adducts to alter physiological proteins. High serum hydroperoxide levels are associated with MCI and AD (Cervellati et al., 2013, 2014a). The OS detected in the serum (high hydroperoxides with low residual antioxidant power) is more pronounced in MCI and AD as compared to vascular dementia (Cervellati et al., 2014b; Table 1), highlighting the specificity of certain lipid peroxidation outcomes in early detection of AD.

Elevated levels of MDA and 4-HNE have been reported in the brains of subjects with MCI or early AD (Keller et al., 2005; Butterfield et al., 2006b; Greilberger et al., 2008; Reed et al., 2008; Lopez et al., 2013; Scheff et al., 2016). Mitochondria isolated from MCI lymphocytes show increased levels of HNE-bound proteins (Sultana et al., 2013). Plasma production of MDA shows a gradation: AD > MCI > healthy controls (Torres et al., 2011) and blood MDA levels have been correlated with the progression of MCI into AD (Baldeiras et al., 2010; Table 1). It should be noted that covalent adducts of 4-HNE are elevated in the brain and body fluids of other neurodegenerative diseases as well including Parkinson’s disease and amyotrophic lateral sclerosis (Di Domenico et al., 2017), necessitating future research on the patterns of MDA and 4-HNE that could distinguish AD from other dementias and neurodegenerative diseases.

The level of F2-isoprostanes, indicating lipid peroxidation, is enhanced in the brain and CSF of MCI and AD patients, but plasma and urinary isoprostanes are normal in AD (Markesbery et al., 2005; Irizarry et al., 2007). A prospective population-based study failed to confirm the association between systemic isoprostanes and the risk of AD (Sundelöf et al., 2009). Despite being touted as “gold standard” biomarker of lipid peroxidation (Butterfield and Halliwell, 2019), the diagnostic use of isoprostanes is tricky because of their non-specificity: isoprostanes have been potential biomarkers for many diseases including obesity, genetic disorders and cancers (Irizarry et al., 2007; Milne et al., 2015).

### Oxidative Damage to Nucleic Acids

Nucleic acid damage also occurs early in AD. Significantly elevated levels of 8OHG and 4,6-diamino-5-formamidopyrimidine have been reported in the post-mortem MCI brains relative to the age-matched controls (Wang et al., 2006). Oxidized RNA nucleoside 8OHG in the neurons of the cerebral cortex is an age-associated phenomenon, but a more prominent RNA damage correlates with MCI and AD (Nunomura et al., 2012). Peripheral leukocytes from MCI and AD patients show enhanced oxidative DNA damage including higher amounts of oxidized purines and pyrimidines relative to the healthy controls (Migliore et al., 2005). Certain nuclear (but not mitochondrial) oxidative phosphorylation genes are upregulated in the hippocampus of MCI patients relative to both AD and normal controls (Mastroeni et al., 2017). What pattern of oxidative damage and gene expression can best distinguish AD at an early stage from other dementia is an open question.

### Reduced Antioxidant Defenses

In addition to oxidative damage, reduced antioxidant defenses have been reported in MCI and early AD (Rinaldi et al., 2003; Baldeiras et al., 2010; Chico et al., 2013). Plasma glutathione levels and antioxidant enzymes such as glutathione peroxidase, catalase, and superoxide dismutase (SOD) are significantly decreased in early AD (Torres et al., 2011; Puertas et al., 2012). Apolipoprotein E4 (APOE4) is the major genetic risk factor in AD. The E4 carriers MCI exhibit significantly reduced plasma SOD activity relative to non-APOE4 carriers (Chico et al., 2013). Plasma SOD activity follows gradation: healthy controls > MCI > AD (Picco et al., 2014). Decreased SOD has also been reported in blood peripheral mononuclear cells of MCI and AD patients (Arce-Varas et al., 2017; Table 1). In other words, reduced antioxidant potential can be detected in both the extracellular and intracellular blood compartments.

Serum analysis of MCI and AD patients have revealed a low residual antioxidant power (Cervellati et al., 2014b). Albumin is considered a major endogenous antioxidant in serum because of its free radical-trapping ability. OS in MCI and AD can increase the serum levels of ischemia-modified albumin (IMA), a form of albumin in which the N-terminal is structurally changed (Du et al., 2019). The levels of selenium, an essential trace element, were found to be lower in both MCI and AD relative to the controls, but plasma selenium was the lowest in the AD group (Rita Cardoso et al., 2014; Table 1). Higher serum levels of gamma-tocopherol, beta-tocotrienol, total tocotrienols, and gamma-tocopherol/cholesterol ratio are associated with a lower risk of MCI or AD in the older adults (Mangialasche et al., 2013). Levels of 5-nitro-gamma-tocopherol, a marker of vitamin E damage, show a significant positive correlation with protein carbonyls, protein-conjugated HNE, and protein-bound 3-nitrotyrosine (Sultana et al., 2013) in MCI and AD (Table 1).

The limited specificity of OS and antioxidant markers must be kept in view. The serum, urine, or CSF concentrations of these biomolecules are associated with several cardiometabolic conditions (Vona et al., 2019) as well. Therefore, OS markers must always be combined with family history and other imaging techniques to detect AD at early stages.

## Natural Antioxidants for Alzheimer’s Disease Prevention and Treatment

### Vitamins

Plasma antioxidant defenses are depleted in MCI and AD (Rinaldi et al., 2003). So, antioxidant intake may be a reliable strategy to prevent or even reverse MCI/AD symptoms. In this regard, vitamin E (tocopherols/tocotrienols) and vitamin C (ascorbate) is considered the scavenging and chain-breaking molecules called direct antioxidants (Mecocci and Polidori, 2012; Polidori and Nelles, 2014). Dietary vitamin E can dictate OS outcomes (Dong et al., 2018). Although vitamin E supplementation cannot stop the progression from MCI to AD, it does delay the onset of AD symptoms (Dysken et al., 2014). Combining vitamin E with vitamin C is better at decreasing F2-isoprostane in the CSF in mild-to-moderate AD than the vitamin E alone (Galasko et al., 2012). The therapeutic importance of the latter observation is yet to be explored.

The risk of AD appears to be decreased in elderly subjects with high plasma levels of vitamin E (tocopherols and tocotrienols; Mecocci and Polidori, 2012; Polidori and Nelles, 2014), but this could be due to a good overall diet rather than vitamin E alone. Almond supplementation on an empty stomach has been found to enhanced memory in animal models of AD (Arslan et al., 2017; Batool et al., 2018). This can partly be explained by high amounts of antioxidants like vitamin E and selenium in almonds (Yada et al., 2011; Arslan et al., 2017). However, a clinical study reported that supplementation with vitamin E and selenium does not ameliorate human dementia (Kryscio et al., 2017). The result of this underpowered study (Kryscio et al., 2017) can be explained by the inclusion of only one gender (men), high loss to follow-up, and short exposure time.

### Polyphenols

Polyphenolic agents, such as curcuminoids found in turmeric, work through multiple pathways and have shown improvements in AD symptoms in animal models (Ahmed and Gilani, 2009, 2014; Ahmed et al., 2014; Khalid et al., 2017), but results of human trials are conflicting (Chen et al., 2018). Gingko Biloba extract contains antioxidant flavonoids among other chemicals. Meta-analyses of human studies have shown promise in AD (Wang et al., 2010; Hashiguchi et al., 2015), but the results of Gingko biloba are far from conclusive (Vellas et al., 2012; Hashiguchi et al., 2015). Like curcumin, the neuroprotection by flavonoid-rich foods may not entirely be due to antioxidant effects since only a limited amount enters the brain. The additional mechanism behind neuroprotection includes flavonoid-induced improvement in brain vascular function (Schaffer and Halliwell, 2012).

Catechins flavonoids are considered the active, therapeutic components of green tea. The ester of epigallocatechin and gallic acid called epigallocatechin-3-gallate (EGCG) is the main bioactive polyphenol in green tea extract that has neuroprotective effects partly owing to its antioxidant activities (Mandel et al., 2011; Mori et al., 2019). Green tea consumption seems to improve cognitive performance in the healthy (Kuriyama et al., 2006) as well as cognitively challenged elderly (Ide et al., 2014). However, the results of a long-term clinical trial of EGCG in the early stages of AD are yet to be published (ClinicalTrials.gov Identifier: NCT00951834). The EGCG dose and frequency needed for AD prevention and/or reversal must be explored further.

Other natural antioxidants tested extensively in animal models but only limitedly in humans for AD include: resveratrol, a polyphenol in grapes and red wine (Rege et al., 2014; Turner et al., 2015); blueberry extract (Papandreou et al., 2009); tannic acid (Mori et al., 2012); and lipoic acid (Siedlak et al., 2009).

## Conclusion

Biomolecules predicting oxidative damage before the onset of clinical systems in AD can help in the diagnosis of this dreaded neurodegenerative disease. AD cannot be detected at an early stage based on oxidative markers alone because of the limited sensitivity and specificity of available options. Among the non-invasive choices, lipid peroxidation (high serum peroxides) holds the most promise in the early detection of AD. It is unlikely, however, that a single non-invasive and cheap biomarker could detect AD at early stages. AD is a complex disease involving multiple pathways. OS is a part of normal aging, but a high OS can be one of the earliest signs of AD. The antioxidants offered to tackle oxidative damage in AD have limited efficacy partly because of the dose, duration, unbalanced monotherapy, and the presence of blood-brain-barrier that does not allow liberal amounts of antioxidants to enter the brain. By the time antioxidants are prescribed in humans, it is already too late. So, a balanced diet and lifestyle modifications can be the only long-term solution to prevent or reverse cognitive impairments associated with the heterogeneous disease that we call AD.

## Author Contributions

JA conceived the idea and wrote the first draft of the manuscript with equal inputs from HJ and HQ.

## Conflict of Interest

The authors declare that the research was conducted in the absence of any commercial or financial relationships that could be construed as a potential conflict of interest.
